# Utilization of Complementary and Alternative Medicine for the Management of Cardiovascular Diseases Among Adults in Ajman, United Arab Emirates

**DOI:** 10.7759/cureus.37394

**Published:** 2023-04-10

**Authors:** Athira Suresh Kumar, Khadija Jalal, Afnan Nurul Aman Shaikh, Swetha Kannan, Jayakumary Muttappallymyalil

**Affiliations:** 1 College of Medicine, Gulf Medical University, Ajman, ARE; 2 Community Medicine, Gulf Medical University, Ajman, ARE

**Keywords:** chronic diseases, adults, hypertension, utilization, alternative medicine, complementary medicine

## Abstract

Background: As the general population is expanding their choices regarding healthcare, many are opting for complementary and alternative medicine (CAM) in addition to or instead of conventional modes of treatment, for the management of various health conditions.

Aims: This study investigated the utilization of CAM for the management of various cardiovascular diseases as well as its risk factors among the adult population in Ajman, UAE.

Materials and methods: The study was conducted upon receiving approval from the Institutional Review Board (IRB). This cross-sectional study was conducted by administering an interviewer-administered questionnaire, consisting of three domains aimed at assessing the sociodemographic features and use of CAM and factors associated with the use among the respondents. A total of 414 responses were collected from adults residing in Ajman, UAE, who consented to participate in the study. A chi-square test was performed on Statistical Product and Service Solutions (SPSS) (IBM SPSS Statistics for Windows, Version 27.0, Armonk, NY) to assess the association between the use of CAM and factors. Statistical significance was set to p ≤ 0.05.

Results: Out of 414 participants in the study, 57% of the participants used CAM before, while 43% of the participants never used CAM. Among the CAM users, 23% used it for anxiety and stress, 7.6% utilized it for the management of hypertension, 3.3% used it for high cholesterol, 3.1% for obesity, 1.9% for chronic kidney disease, 0.9% used it for diabetes mellitus, 0.5% used it for stroke, and 0.5% used it for heart failure.

Conclusions: From the results of the study, it can be concluded that the majority (57%) of the participants have used CAM before. Most of the participants utilized CAM to manage chronic conditions (81.9%).

## Introduction

Complementary and alternative medicine (CAM) is a term used to describe techniques that utilize medicinal items and practices that are not a part of allopathic or evidence-based medicine practice for certain disorders. “Complementary medicine” is said to be used when these techniques are used in addition to conventional therapy. Meanwhile, when they are used instead of conventional treatments, it is known as “Alternative medicine”. Modalities of CAM include categories with a wide range of treatment options, but the most commonly used include homeopathy, ayurveda, acupuncture, wet and dry cupping therapy, yoga, meditation and breathing techniques, herbal medicine, traditional healing, massage, chiropractic therapy, reiki, and Chinese medicine [1].

According to the World Health Organization (WHO) [2], cardiovascular diseases (CVD) are one of the leading causes of death globally as of 2020. CVD are conditions [3] that affect the blood vessels and/or the heart. They are most commonly associated with the increase of fat deposits in arteries, a condition known as atherosclerosis, and a greater risk of forming blood clots that could block these arteries. This results in reduced flow of oxygen-rich blood to the heart muscles or even the brain, causing heart attacks, heart failure, and strokes. CVDs are extremely dangerous as they can cause damage to vital organs like the brain and kidneys. Risk factors of CVDs are hypertension, high cholesterol, diabetes, smoking, and obesity. Studies have shown that even stress and anxiety predispose to CVD [4,5]. Hence, it is important to manage not just the disease itself but also the risk factors that lead to the disease.

With the increased expense and possible side effects, individuals may lean towards the use of complementary and/or alternative treatments for their CVDs. CAM modalities such as meditation, cupping, and others have also proven to be cardioprotective by reducing stress, and anxiety, lowering blood pressure, cholesterol, and even blood sugar [6-9].

Therefore, the present study aims at determining the use of CAM among the adult population. The different literature that is available in the UAE demonstrates that no significant research has ever been done to evaluate CAM usage among adults, especially for the management of CVD. When the research outcome is disseminated, it will help to increase public health as well as improve productivity. The results of this study are important in understanding the potential purposes of CAM and raise the question of whether healthcare professionals should start widening their choices of prescribing treatment. It will help guide policymakers to understand where to focus efficiently. Hence, they can apply appropriate strategies that can elevate the quality of care and client satisfaction. The result of this research will prove useful to healthcare researchers in the future, as it opens a door for further research. Even healthcare educators can rely on the research as a source of literature through which they can educate the common population regarding CAM. The conclusion of the research can help raise awareness among the public regarding the different treatment or management options available for various CVDs and their risk factors.

## Materials and methods

The study followed a cross-sectional design. It was conducted among the adult population residing in Ajman, United Arab Emirates (UAE). An interviewer-administered questionnaire was used to collect data from those adults who fit in the inclusion criteria.

Inclusion criteria

The study was conducted among adults (>18) living in Ajman, UAE. Included only those adults who had signed written consent to participate in the study. Individuals of all genders and nationalities were included.

Exclusion criteria

Individuals who had not given consent to participate in the study were excluded.

Sample size calculation

The sample size was estimated using the formula for cross-sectional study design.

$n=\frac{z^{2}pq}{L^{2}}$

Since we have taken the confidence interval (CI) as 95%, the standard deviation (Z) = 1.96

p = 0.284~0.28 (A previous study done in Abu Dhabi showed that 28.4% had good knowledge about CAM.) [10]

$q = 1- 0.28 = 0.72$

$L = 0.05$

$n=\frac{1.96^{2} \times0.28\times 0.72}{0.05^{2}} = \frac{0.80}{0.0025} = 320$

320 + 10% no response = 320 + 32 = 352 participants ~ 400 participants using convenience sampling technique.

The questionnaire comprised three domains. The first domain collected the sociodemographic characteristics of the participants such as age, gender, nationality, education level, marital status, employment status, field of work, height, and weight. The second and third domains assessed the use of CAM among the participants and the factors associated with it along with the disorders it was used for. A thorough literature search and review were used for guiding the preparation of the questionnaire.

The content was validated by three public health experts. Their suggestions were incorporated, and a pilot study was conducted using the prepared questionnaire for assessing the feasibility, time taken to fill the questionnaire, and for assessing comprehension of the questionnaire. Necessary modifications were made so that the questionnaire was simple and easy to answer yet yielded accurate data.

The research proposal was submitted to the Institutional Review Board (IRB) for approval. The study was conducted upon receiving approval from the IRB (IRB/COM/STD/72/APRIL-2022).

Sampling was done from various residential buildings, shopping malls, taxi stands, hospitals as well as universities located in Ajman, UAE. Written informed consent from the participants was taken along with their signatures. After obtaining the consent, the purpose of the study was described in detail to each participant and the questionnaire was administered to the participants individually. The questionnaire was thereafter reviewed by the interviewer for completion. The raw data from the survey was entered into an Excel spreadsheet. Then it was transferred to Statistical Product and Service Solutions (SPSS) (IBM SPSS Statistics for Windows, Version 27.0, Armonk, NY). Descriptive statistics like frequency and percentage were used. Inferential statistics such as the chi-square test and logistic regression were performed. The association between dependent and independent variables was tested using the chi-square test and a P-value that was less than or equal to 0.05 was considered statistically significant.

## Results

A total of 414 participants were described according to sociodemographic characteristics as shown in Table 1. The age of the participants was categorized into three groups; less than or equal to 24, 25 to 40, and greater than 40 years of age. The majority of the participants were ≤ 24 years (227, 54.8%). The mean age was 29.41 years. The ages of participants ranged from 18 to 70 years old. Among 414, 245 (59.2%) were females and 169 (40.8%) of them were males. According to the WHO classification of regions, the nationalities of the participants were categorized into the South-East Asian region, Eastern Mediterranean region, and others. The majority of the participants belong to the South-East Asian Region (266, 64.3%). Based on their highest level of education, the participants were distributed as those who have an education of high school and below and undergraduate and above. Most of the participants had an education level of undergraduate and above (279, 67.4%). According to marital status, participants were classified as single (272, 65.7%) and married (142, 34.3%). In terms of employment status, employed (188, 45.4%) and unemployed (226, 54.6%). According to International Labour Organization (ILO) classification, the field of work was categorized into white-collar jobs (86, 48.3%), blue-collar jobs (21, 11.8%), and others (71, 39.9%). The participants were also grouped in terms of their BMI into underweight (26, 6.4%), normal weight (227, 56%), overweight (121, 29.9%), and obese or greater (31, 7.7%). The mean BMI is 24.29 and the range is 15.24-42.06.

**Table 1:** Distribution of participants according to socio-demographic characteristics (N= 414) * missing values

Sociodemographic characteristics	Group	No.	%
Age group (in years)	≤24	227	54.8
	24-40	90	21.7
	>40	97	23.4
Gender	Male	169	40.8
	Female	245	59.2
Nationality	South East Asia region	266	64.3
	Eastern Mediterranean region	91	22.0
	Others	57	13.8
Highest educational level	Highschool or below	135	32.6
	Undergraduate or above	279	67.4
Marital status	Single	272	65.7
	Married	142	34.3
Employment status	Employed	188	45.4
	Unemployed	226	54.6
Field of work*	White collar jobs	86	48.3
	Blue collar jobs	21	11.8
	Others	71	39.9
BMI*	Underweight	26	6.4
	Normal weight	227	56.0
	Overweight	121	29.9
	Obese or greater	31	7.7

**Figure 1:**
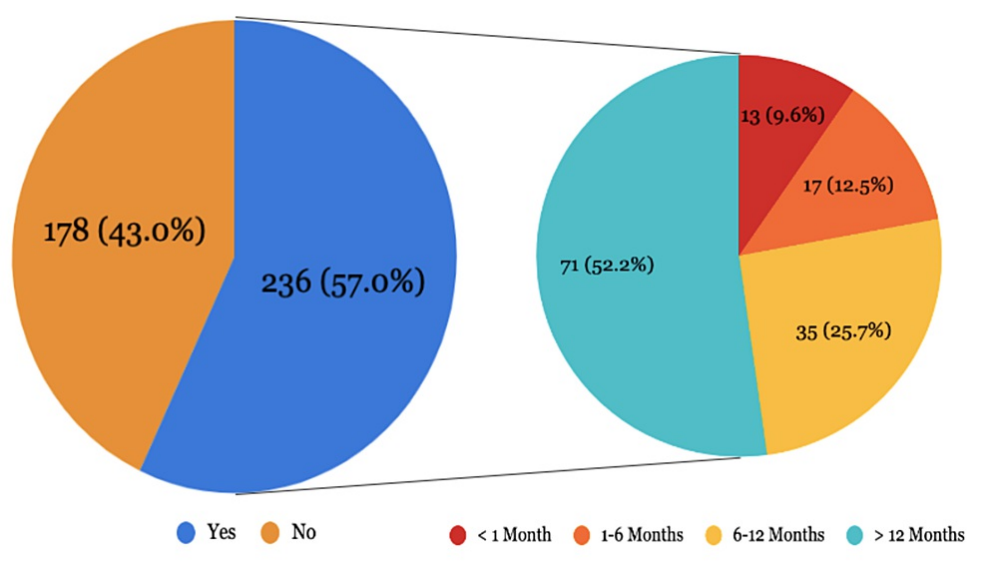
Distribution of participants according to visit to CAM practitioner (N=414) CAM: complementary and alternative medicine

Figure 1 shows that out of the 414 participants in our study, 236 participants (57%) have visited a CAM practitioner. Out of these, the last visit of 13 participants (9.6%) was within the past one month, 17 participants (12.5%) had their last visit within six months, 35 participants (25.7%) within the last one year, and 71 participants (52.2%) visited CAM practitioner more than a year ago.

**Figure 2:**
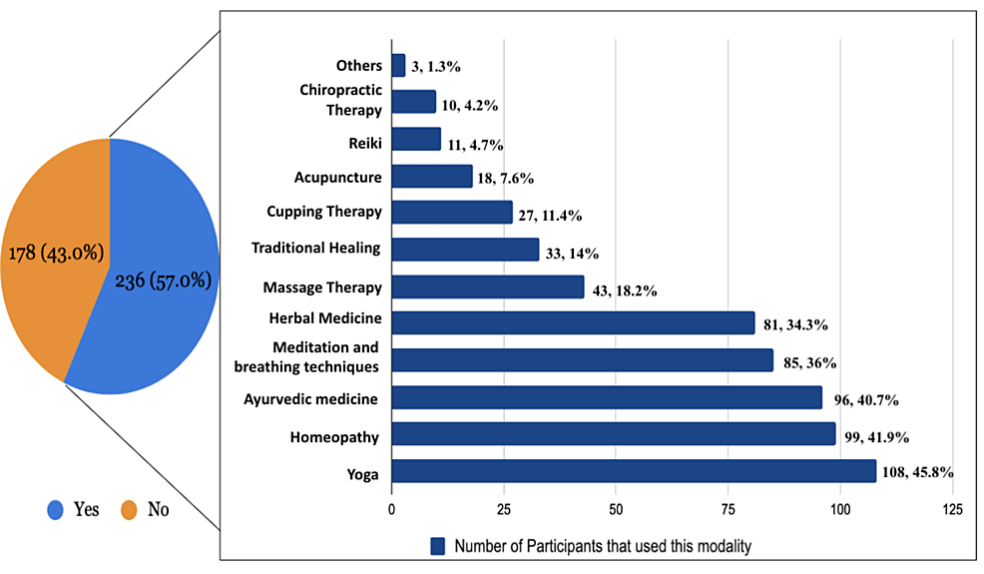
Distribution of participants according to use of CAM and modality of CAM used CAM: complementary and alternative medicine

Figure 2 shows that out of 414 individuals, 236 (57.0%) reported having utilized some type of complementary or alternative therapy, whereas 178 (43.0%) reported having never done so. Figure 2 illustrates the distribution of participants according to the modality of CAM used. Out of the 414 participants, a majority of respondents have tried yoga (n=108, 45.8%) followed by homeopathy (n=99, 41.9%). Ninety-six participants have used ayurvedic medicine (40.7%), 85 have tried meditation and breathing techniques (36%), 81 participants have practiced herbal medicine (34.3%), and 43 participants have tried massage therapy (18.2%). Traditional healing was utilized by 33 participants (14%), cupping therapy by 27 (11.4%), acupuncture by 18 participants (7.6%), and chiropractic therapy was used by 10 respondents (4.2%). Three participants utilized other modalities of CAM.

**Figure 3:**
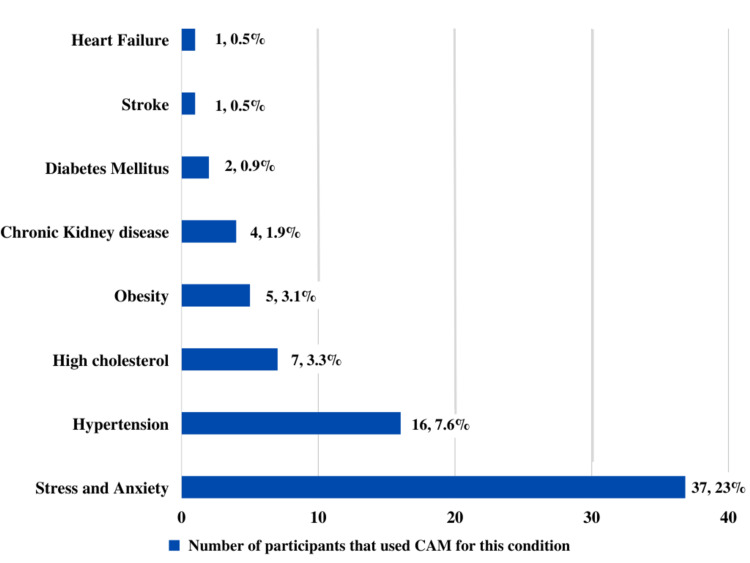
Distribution of participants according to conditions they used CAM for CAM: complementary and alternative medicine

Figure 3 illustrates that among the 236 participants that used CAM, 37 (23%) used it for the management of stress and anxiety issues, 16 (7.6%) used it for hypertension, seven (3.3%) used it for cholesterol, five participants used CAM for obesity (3.1%), and the rest for other CVDs. The rest of the participants used CAM for other conditions such as body pain, cold, skin conditions, menstrual or hormonal issues, and others.

**Figure 4:**
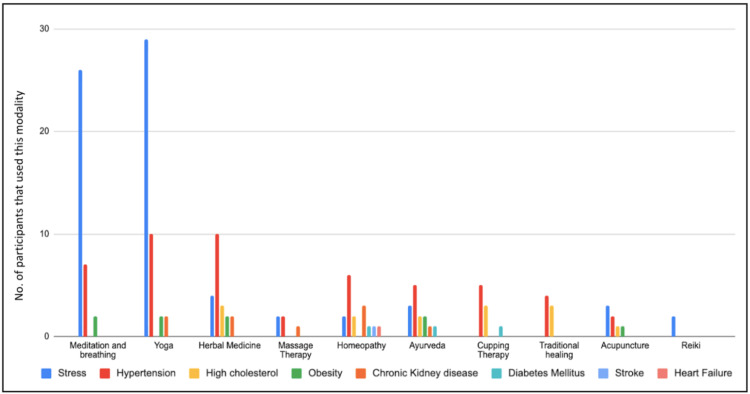
Distribution of participants according to the modality they used for each condition

Figure 4 shows the modalities that the participants used for managing their condition. It was reported that 26 (70.3%) participants used meditation and breathing techniques, 29 (78.4%) used yoga, four (10.8%) used herbal medicine, three (8.1%) used acupuncture, and two (5.4%) used reiki, homeopathy, and massage therapy for dealing with stress and anxiety. For the management of hypertension, 10 (62.5%) participants used yoga and herbal medicine, seven (43.8%) participants used meditation and breathing techniques, six (37.5%) participants used homeopathy, five (31.3%) participants used cupping therapy and ayurvedic medicine, four (25%) participants used traditional healing, and two participants used massage therapy and acupuncture. Three (42.9%) participants used herbal medicine, traditional healing, and cupping therapy for treating high cholesterol, two (28.6%) participants used homeopathy and ayurvedic medicine, and one (14.3%) participant used acupuncture. Obesity was managed by meditation and breathing techniques, yoga, Ayurveda, and herbal medicines by two (40%) participants. One (20%) participant used acupuncture to manage the same. Three (75%) participants used homeopathy while two (50%) participants used yoga and herbal medicine for the management of chronic kidney disease. One (25%) participant used massage therapy for its treatment. Homeopathy, Ayurveda, and cupping therapy were used for the treatment of diabetes by two (100%) participants. One (100%) participant each used homeopathy for managing heart failure and stroke.

To determine the association between socio-demographic characteristics and the use of CAM among the participants, cross-tabulation was done, as seen in Table 2. It showed that 125 (55.1%) participants who were ≤24 years of age, 52 (57.8%) participants between the age of 24-40, and 59 (60.8%) participants older than 40 utilized CAM. Ninety-five (56.2%) male participants and 141 (57.6%) female participants have used CAM before. It was observed that 157 (59%) respondents belonging to the Southeast Asian region, 51 (56%) participants from the Eastern Mediterranean region, and 28 (49.1%) from other regions have utilized CAM before. Sixty-nine (51.1%) respondents having an educational level of high school or below and 167 (59.9%) having an educational level of undergraduate or above have used some form of CAM in the past. It was observed that 147 (54%) of the single participants and 89 (62.7%) of the married participants have used CAM before. Among the employed participants, 115 (61.2%) of them have used CAM while 121 (53.5%) unemployed respondents utilized CAM. Fifty-one (59.3%) participants engaged in white-collar jobs, 11 (52.4%) in blue-collar jobs, and 47 (66.2%) in other jobs have used some form of CAM before. According to BMI classification, 16 (61.5%) underweight participants, 136 (59.9%) normal-weight participants, 65 (53.7%) overweight participants, and 15 (48.4%) obese participants have reported using CAM before. The chi-square test showed no significant association between any of the sociodemographic characteristics of the respondents and the use of CAM (p>0.05).

**Table 2:** Association between socio-demographic characteristics and use of CAM among the participants (N=414) * missing values CAM: complementary and alternative medicine

Sociodemographic characteristics	Group	Ever used CAM				P value
		Yes		No		
		No.	%	No.	%	
Age group (in years)	≤24	125	55.1	102	44.9	0.623
	24-40	52	57.8	38	42.2	
	>40	59	60.8	38	39.2	
Gender	Male	95	56.2	74	43.8	0.787
	Female	141	57.6	104	42.4	
Nationality	South East Asia region	157	59	109	41	0.383
	Eastern Mediterranean region	51	56	40	44	
	Others	28	49.1	29	50.9	
Highest educational level	Highschool or below	69	51.1	66	48.9	0.092
	Undergraduate or above	167	59.9	112	40.1	
Marital status	Single	147	54	125	46	0.092
	Married	89	62.7	53	37.3	
Employment status	Employed	115	61.2	73	38.3	0.118
	Unemployed	121	53.5	105	46.5	
Field of work*	White collar jobs	51	59.3	35	40.7	0.457
	Blue collar jobs	11	52.4	10	47.6	
	Others	47	66.2	24	33.8	
BMI*	Underweight	16	61.5	10	38.5	0.482
	Normal weight	136	59.9	91	40.1	
	Overweight	65	53.7	56	46.3	
	Obese or greater	15	48.4	16	51.6	

## Discussion

In the current study, more than half of the respondents claimed to have used some form of CAM where yoga was most commonly practiced. Similarly, in a study conducted in Abu Dhabi [10], about 95% of the participants had reported utilizing CAM practices and the majority utilized herbal medicine. Results from a study conducted in South Korea [11] showed that a majority of participants (79%) have used one CAM form or the other in their lifetime. These similarities are due to CAM and Korean medicine coexisting in the South Korean healthcare system being equally identified socially and legally.

A majority of respondents in the present study have tried yoga. In contrast, the study conducted in Abu Dhabi [10] showed the most common practice of CAM to be herbals, followed by the use of vitamins, honey products, relaxation, praying, cupping, and meditation. This variation can be attributed to the fact the Abu Dhabi study had a large number of Local Emirati participants and the UAE has a rich and vibrant collection of natural medicinal herbs that have been used traditionally for several generations for the treatment of several illnesses which makes the locals progressively incline towards traditional and herbal methods.

The present study reported that 23% of participants used CAM for the management of stress and anxiety while very few used CAM for the management of hypertension, high cholesterol, diabetes, and others. Another study conducted in the UAE [12] showed that 39.3% used CAM to control diabetes. This difference in results may be because the study was done only among the diabetic population in contrast to the current study which was among the general adult population.

There may be various reasons for opting to use CAM for managing CVD such as unsatisfactory or inefficient results from conventional modes of treatment, unwanted side effects of allopathic medicine, recommendation from family and friends, affordability, and accessibility of certain CAM modalities over other choices.

CAM usage, in the current research, was maximum among participants older than 40 years followed by 24-40 year-olds. Similarly, a study conducted in Korea [11] showed that CAM use was more widely seen among the groups: 40-59 years and over 60 years of age. This can be attributed to the preference for traditional and herbal medicine amongst the older generation. But in Abu Dhabi [10] maximum CAM usage was among participants belonging to the 18-44 years age group. This difference could be due to the wide age categorization done in the Abu Dhabi study.

There was no significant association between the gender and use of CAM in the present study. But a trend was observed where female respondents (141, 57.6%) reported using CAM more than male respondents (95, 56.2%). In a study conducted in Malaysia [13], a greater percentage of female respondents had utilized CAM in their lifetime. Similarly, a study conducted in Norway [14] showed greater CAM usage among females than males. In a US study [15], a significantly higher percentage of CAM usage was seen among females (52%) than among males (44.3%). This similarity can be explained by the possibility that females use various CAM modalities to manage menopause and menstrual symptoms. Females may generally lean toward the utilization of CAM as these don’t require being hospitalized or admitted unlike conventional treatments, hence it saves time.

A significant association between the nationality of participants and the use of CAM was not seen in the current study, but a higher percentage of CAM usage was observed among participants of South East Asian origin (59%). In a study conducted in the US [16] among adults with moderate mental distress, Asians (44.7%) and others (46.8%) had maximum utilization of CAM modalities, while African-Americans (24.3%) had the lowest level of CAM usage. The similarity is probably due to sociocultural similarities among Asians residing in different parts of the world.

The present study showed no significant association between the educational level of the respondents and the use of CAM among them, but a higher percentage of CAM usage was noticed among the participants whose education level was undergraduate or above (59.9%). These results are in line with those from a study conducted in Europe [17], where a higher educational level was associated with greater CAM usage. This can be because those individuals having higher education status is likely to have a better socioeconomic status which allows them to assess and utilize holistic methods of CAM by combining it with conventional therapy.

There was no significant association between the marital status of the respondent and the use of CAM in the current research. However, a greater prevalence of CAM usage is observed among married participants than single respondents. Similarly in the US [18], married individuals were more likely to utilize CAM. This can be attributed to the diffusion of traditional and family-bound values and practices between the partners and their families.

A greater percentage of underweight respondents (BMI < 18.5) in the present research used CAM. However, this finding was not significant. A study conducted in the US [19] showed increased use of CAM among normal-weight and underweight participants with chronic neck pain. In contrast, a study conducted among cancer survivors in the US [20] showed that overweight patients used CAM more as compared to individuals who were normal weight and underweight. The variation can be due to the differences in the sample population used in the two studies.

Limitations

- A cross-sectional study provides only a snapshot of the situation in the sample.

- Data were self-reported. Favorable responses from the participants may lead to an overestimation of the results.

- The generalizability of findings is limited as participants were recruited only from Ajman Emirate.

## Conclusions

To conclude, more than half of the respondents had used some form of CAM before. Among them, a good amount of individuals used CAM for the management of stress and anxiety, others utilized it for the management of hypertension, high cholesterol, obesity, chronic kidney disease, diabetes mellitus, stroke, and heart failure. Sociodemographic factors were not significantly associated with use. Hence, a majority of the participants utilized CAM for the management of CVD and their risk factors, so there is a need to promote awareness regarding the alternative options that are available which can be used instead of or along with evidence-based medicine techniques.
